# Triumph Over Adversity: A Comprehensive Case Series on Successful Pregnancy Outcomes in Antiphospholipid Antibody (APLA)-Positive Patients

**DOI:** 10.7759/cureus.59088

**Published:** 2024-04-26

**Authors:** Manju Mathesan, Shanthi Ethirajan

**Affiliations:** 1 Obstetrics and Gynecology, Saveetha Institute of Medical and Technical Sciences, Chennai, IND

**Keywords:** lupus anticoagulant antibodies, low molecular weight heparin (lmwh), catastrophic antiphospholipid antibody syndrome (caps), spontaneous abortion, abortion, successful pregnancy, antiphospholipid antibody (apla)

## Abstract

The intricate relationship between antiphospholipid antibody (APLA) syndrome and pregnancy outcomes challenges the prevailing notion of inevitable reproductive complications associated with APLA. The introduction provides a comprehensive overview of APLA, its autoimmune thrombophilic nature, and its common association with adverse pregnancy outcomes, emphasizing the need for a nuanced understanding. Here we discuss five case reports to showcase diverse scenarios, each highlighting successful pregnancies in APLA-positive patients, thereby contributing valuable insights into the management of this complex condition. The cases span various clinical presentations, patient demographics, and therapeutic approaches, emphasizing the heterogeneity of APLA-positive pregnancies and the importance of personalized care. The discussion delves into the broader context of APLA's impact on pregnancy, emphasizing recurrent miscarriage and venous thromboembolism (VTE) as severe complications. It underscores the significance of pre-conceptional counseling, a multidisciplinary approach, and regular antenatal monitoring in managing APLA-positive pregnancies. The identification of commonalities among the cases provides a basis for recognizing mitigating factors that contribute to positive outcomes, offering valuable guidance for healthcare providers. The series acknowledges the existence of catastrophic antiphospholipid syndrome (CAPS) and underscores the importance of early recognition and intervention in high-risk cases. Despite the challenges posed by APLA, the cases in the series offer a ray of hope by showcasing instances of successful pregnancies, attributing positive outcomes to optimized therapeutic interventions and vigilant antenatal care. In conclusion, the case series serves as a valuable resource for healthcare professionals, researchers, and policymakers, providing a nuanced perspective on APLA-positive pregnancies. By synthesizing diverse experiences and outcomes, the series contributes to the ongoing dialogue surrounding optimal management strategies, ultimately aiming to improve the quality of care for individuals facing the unique challenges posed by APLA during their reproductive journey.

## Introduction

Antiphospholipid antibody (APLA) syndrome is a systemic disorder diagnosed through the presence of lupus anticoagulant (LAC) and anti-cardiolipin antibodies (ACA), often linked to venous and/or arterial thrombosis or complications during pregnancy. This autoimmune thrombophilic condition involves the antibodies in the blood attacking phospholipid-binding proteins rather than the phospholipid itself and is commonly associated with other autoimmune diseases, particularly systemic lupus erythematosus (SLE). Women are disproportionately affected, with female to male ratio of 5:1 [1].

These antibodies stimulate the activation of endothelial cells, monocytes, and platelets, resulting in excessive production of tissue factor and thromboxane A2, along with complement activation. Pregnancy, already presenting as a hypercoagulable state, undergoes further complications due to changes in the hemostatic system, leading to issues such as recurrent miscarriage, preterm labor, fetal growth restriction (FGR), fetal distress, prematurity, pre-eclampsia/eclampsia, HELLP (hemolysis, elevated liver enzymes, and low platelets) syndrome, and placental insufficiency [2].

In some instances, a more severe form known as catastrophic antiphospholipid antibody syndrome (CAPS), or Asherson syndrome, can manifest. This condition is characterized by rapid and progressive thromboembolism involving three or more organ systems simultaneously, resulting in high maternal mortality. While APLA is recognized as a significant risk factor for adverse pregnancy outcomes, this case series explores a subset of patients who have achieved successful reproductive outcomes, challenging the prevailing notion that APLA inevitably leads to reproductive complications. The series aims to illuminate the intricate interplay between antiphospholipid antibodies and positive pregnancy outcomes, identifying commonalities, mitigating factors, and effective management strategies.

By presenting diverse cases encompassing various aspects of APLA, maternal demographics, and therapeutic approaches, the series seeks to unravel the complexities surrounding successful pregnancies in APLA-positive individuals [3]. It aims to provide a nuanced perspective for clinicians, contributing to improved clinical care and better outcomes for individuals facing unique challenges posed by APLA during their reproductive journey. This comprehensive case series is intended to serve as a valuable resource for healthcare professionals, researchers, and policymakers, fostering a deeper understanding of the factors influencing successful pregnancies in APLA-positive patients. Through the synthesis of collective experiences, the series aims to contribute to the ongoing dialogue surrounding antiphospholipid syndrome (APS) and pregnancy, ultimately paving the way for enhanced clinical care and improved outcomes [4].

## Case presentation

Case report 1

A 30-year-old woman, gravida 4, para 1, living 0, abortus 2, death 1, had a history of previous cesarean section, hypothyroidism, and a miscarriage at 11 weeks of gestation five years ago. Additionally, she had experienced a hypertensive disorder during her second pregnancy and intrauterine fetal demise at seven months of gestation two years back. She presented at eight weeks of gestation for a routine antenatal checkup at the Obstetrics and Gynecology Department, Saveetha Medical College and Hospital. Due to her adverse obstetric history, an APLA profile was conducted, revealing a positive result for LAC. At 13 weeks, the patient was commenced on a daily dose of 75 mg of aspirin (Ecospirin) and 60 mg/0.6 ml subcutaneous injection of enoxaparin once daily. Extensive counseling regarding maternal and fetal risk factors associated with APLA syndrome was provided, and she underwent regular antenatal follow-up. All investigations, including hematocrit, coagulation profiles, proteinuria, and USG scans, remained within normal limits throughout her pregnancy, done at an interval of each trimester. At 36 weeks of gestation, aspirin was discontinued, and subsequent routine investigations continued to yield normal results. Growth scans, including amniotic fluid index (AFI) Doppler, indicated normal fetal growth and well-being. However, at 37 weeks and two days, the patient presented with abdominal pain and impending scar tenderness, necessitating an emergency cesarean section as there might be a chance for uterine rupture. She successfully delivered a live, term baby girl weighing 2.8 kg, appropriate for gestational age (AGA), with appearance, pulse, grimace, activity, and respiration (APGAR) scores of 8 and 9 at one and five minutes, respectively. The post-operative period was uneventful, and the patient's vital signs remained stable. Low molecular weight heparin (LMWH) was initiated 12 hours after cesarean section. Follow-up coagulation profiles and complete blood counts continued to demonstrate normal results. The summary of the case is presented in Table 1.

**Table 1:** Summary of case 1 APLA: Antiphospholipid antibody; APGAR: Appearance, pulse, grimace, activity, and respiration; LMWH: Low molecular weight heparin

	Age	Obstetric Score	Previous Obstetric History	APLA Test	Antepartum Medications	Intrapartum Period	Postpartum Period
Case 1	30 years	G_4_P_1_L_0_A_2_D_1_	1) Previous cesarean section; 2) Hypothyroidism; 3) A past miscarriage at 11 weeks of gestation five years ago; 4) Hypertensive disorder during her second pregnancy; 5) Intrauterine fetal demise at the seventh month of gestation two years back	Positive for lupus anticoagulant	1) Tab. Ecosprin 75 mg once daily; 2) Inj. Enoxaparin 60 mg/0.6 ml s/c once daily	At 37 weeks and two days, the patient delivered a live, term baby girl, weighing 2.8 kg, appropriate for gestational age, and APGAR scores of 8/10 and 9/10	Post-operatively LMWH was at 12 hours and continued till six weeks postpartum

Case report 2

A 26-year-old woman, gravida 3, para 1, living 1, abortus 1, had experienced a spontaneous abortion at 10 weeks of gestation in her first pregnancy. Her second pregnancy was complicated by preterm indicated cesarean section at 28 weeks due to intrauterine growth restriction (IUGR) and severe oligohydramnios. The infant, a baby girl weighing only 600 g, required admission to the Neonatal Intensive Care Unit (NICU) for two months but was discharged in an improved condition. During her current pregnancy, the patient was evaluated for APLA syndrome at eight weeks of gestation, with results showing a strongly positive LAC. Subsequent investigations including hematocrit, coagulation profiles, and proteinuria remained within normal limits, done at an interval of each trimester. She was commenced on tablet Ecosprin 75 mg once daily and injection enoxaparin 60 mg/0.6 ml s/c once daily for APLA syndrome, along with tablet labetalol 100 mg twice daily for chronic hypertension associated with proteinuria (protein 1+) diagnosed at 12 weeks of gestation. Ophthalmology evaluation was done regularly in each trimester, which revealed no evidence of hypertensive retinopathy during the fundus examination. She also had a history of hypothyroidism for the past five years and was managed with replacement therapy of 37.5 mcg once daily. At 28 weeks of gestation, the patient was admitted due to asymmetrical IUGR with oligohydramnios with a maximum vertical pocket of 1.7 cm, and Doppler studies were normal. Steroids were administered and regular antenatal follow-up was continued. As the pregnancy progressed, aspirin was discontinued at 36 weeks and injection Clexane was stopped at 37 weeks and four days. Given her previous cesarean and breech presentation, an elective cesarean section was performed at 38 weeks. The patient delivered a live, term, small for gestational age (SGA) baby girl weighing 2.140 kg, with APGAR scores of 7 and 9 at one and five minutes, respectively. Due to the low birth weight, the baby was admitted to NICU for monitoring. The patient remained stable during the post-operative period, and LMWH was initiated 12 hours after cesarean section. All routine investigations and coagulation profiles were found to be normal. The summary of the case is presented in Table 2.

**Table 2:** Summary of case 2 APLA: Antiphospholipid antibody; APGAR: Appearance, pulse, grimace, activity, and respiration; LMWH: Low molecular weight heparin; IUGR: Intrauterine growth restriction; SGA: Small for gestational age

	Age	Obstetric Score	Previous Obstetric History	APLA Test	Antepartum Medications	Intrapartum Period	Postpartum Period
Case 2	26 years	G_3_P_1_L_1_A_1_	1) Spontaneous abortion at 10 weeks of gestation; 2) Second pregnancy - preterm elective cesarean section at 28 weeks due to IUGR and severe oligohydramnios	Strongly positive lupus anticoagulant	1) Tab. Ecosprin 75 mg once daily; 2) Inj. Enoxaparin 0.6 ml s/c once daily; 3) Tab. Labetalol 100 mg twice daily - for chronic hypertension; 4) Tab. Thyronorm 37.5 mcg once daily - for hypothyroidism	At 38 weeks, the patient delivered a live, term, SGA baby girl weighing 2.140 kg, with APGAR scores of 7/10 and 9/10	Post-operatively LMWH was at 12 hours and continued till six weeks postpartum

Case report 3

A 29-year-old woman, gravida 5, para 4, living 1, dead 3, with a history of four previous uncomplicated vaginal deliveries, presented at eight weeks of gestation. However, she had experienced intrauterine fetal demise in her second, third, and fourth pregnancies at seven months of gestation, occurring five, four, and three years ago, respectively. Due to her adverse obstetric history, the patient underwent evaluation for APLA and tested positive for anti-beta 2 glycoprotein. Subsequent routine investigations including hematocrit, coagulation profiles, proteinuria, and USG scans were all within normal limits, done at an interval of each trimester. She commenced on a daily dose of tablet Ecospirin 75 mg once daily and the injection enoxaparin 60 mg/0.6 ml s/c once daily. The patient remained under regular antenatal follow-up. As she reached 36 weeks, aspirin was discontinued, and at 37 weeks and four days, injection Clexane was also stopped. However, due to fetal distress with late deceleration, an emergency cesarean section was performed at 37 weeks and six days. The patient delivered a live, term baby girl, weighing 2.7 kg, with APGAR scores of 7 and 8 at one and five minutes, respectively. The post-operative period was uneventful, and LMWH was initiated 12 hours after cesarean section. Subsequent coagulation profiles and routine investigations returned within normal limits. The summary of the case is presented in Table 3.

**Table 3:** Summary of case 3 APLA: Antiphospholipid antibody; APGAR: Appearance, pulse, grimace, activity, and respiration; LMWH: Low molecular weight heparin

	Age	Obstetric Score	Previous Obstetric History	APLA Test	Antepartum Medications	Intrapartum Period	Postpartum Period
Case 3	29 years	G_5_P_1_L_1_D_3_	Intrauterine fetal demise with her second, third, and fourth pregnancies	Anti-beta 2 glycoprotein IgM = 30 SMU/ml; Anti-beta 2 glycoprotein IgG = 35 SGU/ml	1) Tab. Ecosprin 75 mg once daily; 2) Inj. Enoxaparin 0.6 ml s/c once daily	At 37 weeks and six days, the patient delivered a live, term baby girl, weighing 2.7 kg, with APGAR scores of 7/10 and 8/10	Post-operatively LMWH was at 12 hours and continued till six weeks postpartum

Case report 4

A 28-year-old woman, with an obstetric score of gravida 7, abortus 6 at seven weeks of gestation, had a poor obstetric history including multiple spontaneous abortions and missed miscarriages. She had a documented history of seizure disorder and was on tablet levetiracetam and tablet carbamazepine for the past seven years. Following the evaluation for APLA, she tested positive for anti-cardiolipin IgG and LAC. Subsequent routine investigations including hematocrit, coagulation profiles, proteinuria, and USG scans were all within normal limits, done at an interval of each trimester. She was initiated on treatment including injection heparin (LMWH) and oral forms of current meds (Wysolone, hydroxychloroquine, Ecospirin, Rantac, folic acid, levetiracetam, and carbamazepine) and remained under regular antenatal follow-up. Experiencing a seizure at 30 weeks of gestation, aspirin was discontinued at 36 weeks, and the injection Clexane was ceased at 38 weeks. However, due to fetal distress, an emergency cesarean section was performed at 39 weeks, resulting in the delivery of a live, term baby girl weighing 3 kg, with APGAR scores of 8 and 9 at one and five minutes, respectively. Her post-operative recovery was uneventful, with LMWH initiated 12 hours after cesarean section. Subsequent coagulation profiles and routine investigations returned within normal limits. The summary of the case is presented in Table 4.

**Table 4:** Summary of case 4 APLA: Antiphospholipid antibody; APGAR: Appearance, pulse, grimace, activity, and respiration; LMWH: Low molecular weight heparin

	Age	Obstetric Score	Previous Obstetric History	APLA Test	Antepartum Medications	Intrapartum Period	Postpartum Period
Case 4	28 years	G_7_A_6_	1) Spontaneous abortion at eight weeks of gestation; 2) Missed miscarriage at 10 weeks; 3) Spontaneous abortion at 10 weeks of gestation; 4) Spontaneous abortion at 17 weeks of gestation; 5) Spontaneous abortion at eight weeks of gestation; 6) Missed miscarriage at 12 weeks of gestation	Positive for anti-cardiolipin IgG and lupus anticoagulant	1) Inj. Heparin (LMWH) 0.6 ml s/c once daily; 2) Tab. Wysolone 7.5 mg once daily; 3) Tab. Hydroxychloroquine 200 mg twice daily; 4) Tab. Ecosprin 75 mg once daily; 5) Tab. Rantac 150 mg twice daily; 6) Tab. Folic acid 5 mg once daily; 7) Tab. Levitiracetam; 8) Tab. Carbamazepine	At 39 weeks, the patient delivered a live, term baby girl, weighing 3 kg, with APGAR scores of 8/10 and 9/10	Post-operatively LMWH was at 12 hours and continued till six weeks postpartum

Case report 5

A 26-year-old, gravida 5, para 1, abortus 4, at nine weeks and six days of gestation, presented to the Outpatient Department with a history of adverse obstetric events, including missed miscarriages and a previous molar pregnancy, all requiring dilation and curettage (D&C). Following evaluation for APLA, she tested positive for beta 2 glycoprotein-1 IgM. Subsequent routine investigations including hematocrit, coagulation profiles, proteinuria, and USG scans were all within normal limits, done at an interval of each trimester. Treatment was initiated with LMWH 0.6 ml s/c once daily, tablet hydroxychloroquine 200 mg twice daily, tablet Ecospirin 75 mg once daily, tablet Rantac 150 mg twice daily, and tablet folic acid 5 mg once daily, with regular antenatal follow-up. As she reached 36 weeks, aspirin was discontinued, and the injection Clexane was stopped at 38 weeks. At 39 weeks, she delivered a live, term baby girl weighing 2.9 kg, with APGAR scores of 7 and 8 at one and five minutes by vaginal delivery. Her post-operative recovery was uneventful, with LMWH initiated 12 hours after delivery. Subsequent coagulation profiles and routine investigations returned within normal limits. The summary of the case is presented in Table 5.

**Table 5:** Summary of case 5 APLA: Antiphospholipid antibody; APGAR: Appearance, pulse, grimace, activity, and respiration; LMWH: Low molecular weight heparin; D&C: Dilation and curettage

	Age	Obstetric Score	Previous Obstetric History	APLA Test	Antepartum Medications	Intrapartum Period	Postpartum Period
Case 5	26 years	G_5_P_1_A_4_	1) Missed miscarriage at two months of gestation; 2) Missed miscarriage at two months of gestation - D&C done; 3) Missed miscarriage at two months of gestation - D&C done; 4) Molar pregnancy one year back - D&C done	Positive for beta 2 glycoprotein-1 IgM	1) Inj. Heparin (LMWH) 0.6ml s/c once daily; 2) Tab. Hydroxychloroquine 200 mg twice daily; 3) Tab. Ecosprin 75 mg once daily; 4) Tab. Rantac 150 mg twice daily; 5) Tab. Folic acid 5 mg once daily	At 39 weeks, the patient delivered a live, term baby girl, weighing 2.9 kg with APGAR scores of 7/10 and 8/10	Post-operatively LMWH was at 12 hours and continued till six weeks postpartum

## Discussion

The most prevalent obstetric manifestation of APLA is a recurrent miscarriage, with approximately 10-20% of recurrent pregnancy loss associated with APS, and 21-56% of SLE patients having secondary APS [3,4]. The Sapporo criteria, as outlined by the International Consensus Statement and American College of Obstetricians and Gynecologists (ACOG), is utilized for APS diagnosis, encompassing various fetal and maternal complications such as preterm delivery, oligohydramnios, prematurity, fetal growth restriction (30-60%), pre-eclampsia (30%), eclampsia, HELLP syndrome, and placental insufficiency [5]. Venous thromboembolism (VTE) represents another severe complication in APS during pregnancy, carrying a 4-5 times higher risk compared to non-pregnant women. In pregnancy, deep vein thrombosis (DVT) often manifests as a left lower extremity clot due to the compression of the left common iliac vein by the enlarging gravid uterus [6]. Pre-conceptional counseling and a multidisciplinary approach play a pivotal role in managing women with APS during pregnancy, outlining risks and appropriate management strategies. Regular antenatal visits are recommended every 2-4 weeks in the second trimester and 1-2 weeks in the third trimester. For cases with a poor obstetric history, evidence of preeclampsia, or growth retardation, USG is advised every 3-4 weeks starting at 18-20 weeks of gestation. Fetal surveillance should commence at 32 weeks of gestation or earlier if uteroplacental insufficiency is suspected, and continue at least weekly until delivery [7].

The comprehensive case series on favorable pregnancy outcomes in APLA-positive patients establishes an invaluable platform for a nuanced exploration of the intricate relationship between APLA syndrome and reproductive success [8]. The presented cases collectively contribute to a deeper understanding of the complexities inherent in managing pregnancies in individuals with APLA, challenging the prevailing narrative of inevitable adverse outcomes. A noteworthy observation from the case series is the diversity in clinical presentations and patient demographics. The spectrum of cases encompasses various manifestations of APLA, ranging from isolated APLA positivity to its association with SLE and other autoimmune conditions [9]. This diversity underscores the necessity for personalized and tailored approaches in managing APLA-positive pregnancies, considering the heterogeneous nature of this patient population [10]. The identification of commonalities among the cases sheds light on potential mitigating factors contributing to successful pregnancy outcomes in APLA-positive individuals. Whether through specific therapeutic interventions, close monitoring, or other factors, recognizing these patterns provides valuable insights for healthcare providers. Furthermore, understanding the factors contributing to positive outcomes enables clinicians to refine their management strategies, thereby improving the overall care of APLA-positive pregnant patients [11].

The discussion also highlights the broader context of APLA's impact on pregnancy and the associated hemostatic changes. The antibodies associated with APLA stimulate the activation of endothelial cells, monocytes, and platelets, resulting in an exaggerated production of tissue factor and thromboxane A2, alongside complement activation. These changes, coupled with the inherent hypercoagulable state of pregnancy, contribute to a range of complications, including recurrent miscarriage, preterm labor, fetal growth restriction, and other obstetric complications [12]. Despite these challenges, the cases in this series offer a ray of hope by showcasing instances of successful pregnancies. This positive outcome may be attributed to various factors, such as optimized therapeutic interventions, vigilant antenatal care, and a multidisciplinary approach involving obstetricians, rheumatologists, and hematologists. Discussing these cases helps elucidate the strategies that have proven effective in navigating the complexities of APLA-positive pregnancies [8-10]. The series also acknowledges the existence of a more severe manifestation, CAPS, emphasizing the importance of early recognition and intervention in high-risk cases. CAPS, characterized by rapid and progressive thromboembolism involving multiple organ systems, underscores the critical need for swift and targeted management to mitigate maternal morbidity and mortality.

"A case series on successful pregnancy outcomes in patients with APLA syndrome" by Gaur et al. [13] details the management strategies such as the use of heparin and low-dose aspirin that resulted in successful outcomes. Chandana et al. [14] in "APLA syndrome in pregnancy and its outcome: a case report" emphasizes the significance of early diagnosis and treatment with anticoagulants. A broader study, "Pregnancy outcomes in antiphospholipid antibody positive patients: prospective results from the AntiPhospholipid Syndrome Alliance for Clinical Trials and InternatiOnal Networking (APS ACTION) Clinical Database and Repository ('Registry')" by Erton et al. [15], points to the benefits of consistent monitoring and adjusted anticoagulant therapy. Similarly, "Successful treatment outcomes in pregnant patients with ANCA-associated vasculitides: a systematic review of literature" by Singh et al. [16] suggests beneficial outcomes through targeted immunosuppressive treatments in similar autoimmune conditions. Farber et al. [17] in "Successful foetal outcome in a patient with primary membranous nephropathy and circulating anti-phospholipid antibodies: a case report" discusses managing complex APS with specific interventions targeted at individual patients leading to a successful pregnancy.

This comprehensive case series on successful pregnancy outcomes in APLA-positive patients not only contributes to the existing body of knowledge on APLA and pregnancy but also serves as a practical guide for clinicians. By synthesizing diverse experiences and outcomes, the series contributes to the ongoing dialogue surrounding optimal management strategies, ultimately improving the quality of care for pregnant women facing the unique challenges posed by APLA during their reproductive journey.

## Conclusions

In conclusion, the diagnosis of APS necessitates a vigilant approach, particularly when assessing women with recurrent pregnancy loss and vascular thrombosis. Initiating LMWH thrombo-prophylaxis and low-dose aspirin promptly upon detecting cardiac activity can contribute to successful pregnancy outcomes, achieving a 70% success rate. Obstetricians should actively screen for anti-cardiolipin and LAC antibodies in cases of recurrent pregnancy loss. Additionally, patients with elevated levels of ACA and beta 2 glycoprotein-1 antibodies face an increased risk of miscarriage. The overarching goal of managing APS is to enhance both maternal and fetal outcomes. A multidisciplinary approach is imperative for optimal patient care. Successful pregnancies can be realized through pre-conceptional counseling, judicious selection of anticoagulants early in pregnancy with minimal risks to both mother and fetus, and vigilant monitoring throughout the entirety of the pregnancy.
